# Velocity-Aided Navigation in GNSS-Denied Environments

**DOI:** 10.3390/s26155006

**Published:** 2026-08-06

**Authors:** Vadym Avrutov, Nadiia Bouraou, Oleg Nesterenko, Sergii Holovach, Oleksii Hehelskyi, Olha Pazdrii

**Affiliations:** 1 Department of Computer-Integrated Optical and Navigation Systems, National Technical University of Ukraine ‘Igor Sikorsky Kyiv Polytechnic Institute’, 03056 Kyiv, Ukraine; nburau@ukr.net (N.B.); o.hehelskyi@kpi.ua (O.H.); o.pazdrii@kpi.ua (O.P.); 2 Inertial Labs UA, 01010 Kyiv, Ukraine; 3 Joint Stock Company ‘ELMIZ’, 02099 Kyiv, Ukraine; golovach.s@meta.ua

**Keywords:** velocity, navigation, latitude, longitude, altitude, gyroscopes, accelerometers, global navigation satellite system, strapdown inertial navigation systems

## Abstract

**Highlights:**

**What are the main findings?**

- An alternative method for aircraft/drone navigation is proposed when the usual SINS/GNSS methods do not work.
- Semi-natural experiments confirmed the accuracy of the alternative method at the level of accuracy of SINS/GNSS methods, especially at intervals of up to 10 min.

**What are the implications of the main findings?**

- The proposed VAN method is quite simple, since it is implemented using MEMS technology.
- The proposed method can be used as a backup method for aircraft navigation.

**Abstract:**

The Velocity-Aided Navigation (VAN) method for determining latitude, longitude, and altitude is proposed when global navigation satellite system (GNSS) signals are unavailable. Currently, GNSS receivers are the primary navigation systems that meet consumer demand for location accuracy. However, GNSS receivers are not autonomous. Strapdown inertial navigation systems (SINSs), unlike GNSSs, are autonomous. Their operating principle is based on double integration of accelerometer output signals. However, they have a significant drawback: SINS errors increase significantly over time. Two approaches are used to improve accuracy. The first involves using expensive, high-precision gyroscopes and accelerometers. The other involves correcting the SINS by integrating it with navigation systems built on physical principles different from those of the SINS. An alternative method, based on VAN and an inertial measurement unit (IMU), for determining navigation parameters is proposed and does not require double integration of accelerometer output signals. Analytical expressions for the errors of the new method are derived. Calculations showed that the errors of the new method are significantly smaller than those of the autonomous SINS. Experimental testing confirmed the calculation results and demonstrated that the errors of the new method are comparable to those of the SINS integrated with GNSS using a Kalman filter. The proposed alternative VAN method for determining latitude, longitude, and altitude can be used independently, as an alternative to GNSS for integration with the SINS, and can also serve as a backup navigation system.

## 1. Introduction

Today, global navigation satellite system (GNSS) receivers are arguably the primary navigation systems, achieving high accuracy in determining location coordinates. Unfortunately, GNSSs are not autonomous navigation systems, as they depend on the operation of a satellite constellation and environmental conditions. This operation can be affected by various natural and artificial factors [1]. Navigation in GNSS-denied environments remains one of the major challenges for autonomous UAV operation. Recent comprehensive reviews demonstrate the broad diversity of current GNSS-denied navigation approaches, including inertial, vision-based, LiDAR, terrain-aided, and multi-sensor fusion methods [2].

Unlike GNSS, strapdown inertial navigation systems (SINSs) are autonomous. Their operating principle is based on double integration of accelerometer readings. Since the accelerometer output signal contains errors in addition to the useful signal, after double integration, the SINS errors increase nonlinearly with time as will be shown below. To correct the SINS operation, they are integrated with navigation systems built on physical principles different from those of SINSs. For example, they are often integrated with GNSSs. However, after such integration, the navigation systems cease to be autonomous. Another way to reduce INS errors is to use expensive, high-precision gyroscopes and accelerometers. However, this significantly increases the cost of such SINSs. Such SINSs are used in space missions or for special purposes.

Aircraft and autonomous systems increasingly operate in GNSS-denied or contested areas, necessitating robust standalone navigation capabilities. In these challenging environments, Velocity-Aided Navigation (VAN) technology comes to the rescue. It is a technique that estimates a vehicle’s position, orientation, and motion by integrating velocity measurements from velocity sensors.

For underwater navigation, Doppler velocity logs (DVLs) are used as velocity meters. Most of the work is devoted to the autonomous underwater vehicle (AUV). For example, ref. [3] describes a small AUV designed to perform research tasks. Its navigation system consisted of an inertial navigation system (INS) integrated with a DVL, a magnetometer, and a pressure sensor (PS). Typically, INS uses the velocity provided by the DVL for underwater operation. In practice, partial DVL measurements occur when bottom keeping is impossible. Then, the DVL cannot correctly estimate the vehicle’s velocity. In these situations, velocity data cannot be integrated into the AUV’s INS. As a result, the INS error will increase over time. To solve this problem, it was proposed to use partially raw DVL data and additional information to determine the AUV’s velocity. This allowed the use of an extended loosely coupled integration (ELC) approach. Implementing ELC requires only software modifications. A six-degree-of-freedom (6DoF) AUV simulation was presented, including all functional subsystems. This simulation allowed us to evaluate the proposed method and demonstrate the benefits of its use.

The article [4] examined the observability of a combined navigation system consisting of an IMU and a DVL. It was shown that, in limited cases, such a combined system is observable. It was shown that DVL parameters such as the scale factor and offset angles can be calibrated without external sources such as GPS or acoustic beacons. The simulation results confirmed the analytical conclusions.

SINS alignment in motion is one of the most challenging tasks for AUVs, especially when no observations are available in the geodetic reference system. A new SINS/DVL alignment method using the Unscented Kalman Filter (UKF) is presented in [5]. The distinctive feature of UKF is that it allows for large initial deviations. The proposed method presents a nonlinear SINS error model. The measurement model is considered under the assumption that large deviations may exist. For the robustness of UKF, knowledge of the measurement noise covariance is of great importance. That is why the a priori covariance matching methods widely used in Adaptive KF (AKF) are extended for use in Adaptive UKF (AUKF). Experimental results showed that the proposed INS/DVL alignment model is effective for any initial heading errors. The paper evaluates the effectiveness of the adaptive filtering methods in terms of the stability of the parameter estimates. It is also shown that the measurement noise covariance can be reliably estimated using AUKF methods, which indicates an improvement in the alignment quality.

In addition to the mentioned Kalman filter variations, INS and DVL measurements from the AUV are integrated using the extended Kalman filter (EKF) [6]. DVL velocity vector estimation depends on the reconstruction of seafloor reflections. A successful reflection is achieved when at least three of the four transmitted acoustic beams are returned. If less than three beams are received, DVL does not provide velocity updates and the INS drift increases with time. In the presence of a limited number of DVL measurements, a hybrid neural-coupled (HNC) approach is proposed for seamless AUV navigation. First, a regression of two or three missing DVL beams is proposed. Together with the measured beams, these beams are included in the EKF. The authors investigated this fusion for weakly and strongly coupled INS/DVL. Experiments conducted in the Mediterranean Sea showed that the proposed approach ensures seamless AUV navigation in situations with a limited number of beam measurements.

Complex hydrographic conditions and the absence of GNSS signals underwater lead to a loss of INS/DVL navigation accuracy [7]. A hybrid navigation method that combines real-time hydrographic information is proposed. This hybrid navigation method is based on physical principles and real-world data. A multi-layered error suppression system is created. At the physical layer, conductivity–temperature–depth (CTD) sensors are used. These sensors are designed to construct sound velocity profiles in real time. Ray tracing based on Snell’s law is applied to correct DVL sound velocity scale factors and geometric errors caused by refraction. A hybrid CNN-MLP network, based on the obtained data, intelligently estimates and compensates for residual perturbations in the velocity of ocean currents that remain after physical corrections. Sea trial results validate the proposed hybrid navigation method. The physical layer effectively corrects acoustic geometric errors, reducing the root mean square velocity error by approximately 35%. A layer based on real data compensates for dynamic residual environmental fluctuations. This reduces the root mean square velocity error by approximately 60%, and suppresses positioning error by approximately 80%. This study confirms that the combination of physical models with machine-learning-based correction effectively overcomes drift caused by DVL bottom grip loss and time-varying currents. This enables high-precision and long-term UAV operations.

SINS integrated with DVL is the primary navigation solution for UAVs [8]. Unfortunately, when the distance between the UAV and the seabed is beyond the operating range, DVL cannot obtain the velocity relative to the seabed. This often occurs when UAVs navigate in the mid-ocean layer. For integrated SINS/DVL, the unknown current velocity is coupled with the measured velocity error. This leads to a decrease in positioning accuracy. To address this issue, the influence of the unknown coupled current velocity is analyzed in terms of filter observability. An integrated SINS/DVL/virtual velocity navigation method is proposed as a solution. The virtual velocity is constructed from the velocity extracted from the IMU and DVL. This virtual velocity is used as an auxiliary measurement for the Kalman filter. Using the virtual velocity, the current velocity can be easily separated from the measured SINS velocity error. As shown by the simulation and experimental results, the proposed method can effectively improve the positioning accuracy compared to the classical SINS/DVL integration method.

Undoubtedly, navigation accuracy is of great importance for UAVs, which are designed for the marine environment and resource exploration [9]. INS, DVL, and PS have efficient and convenient configurations, and therefore they are preferable for integration onboard underwater vehicles. For example, speed measurement using DVL leads to a reduction in the accumulated error of INS. The traditional solution for such INS/DVL/PS integration is filter-based integration. The authors propose a factor graph optimization (FGO) framework for INS/DVL/PS navigation system integration. Experience shows that, due to harsh sea conditions and range limitations, DVL signals often contain outliers and glitches. This leads to rapid position drift and error accumulation in the navigation system. To address this phenomenon, the authors propose an outlier detection scheme based on an improved interquartile range method. This method combines a sliding window and dynamic adjustment of the propeller revolutions per minute (RPM) threshold. Furthermore, the authors propose a DVL speed prediction method based on a nonlinear least squares (NLS)–Transformer–LSTM model. The authors refine the RPM model using NLS and use it as one of the key features in the prediction model. Finally, the constructed system is compared with several classical Kalman filters both through simulations and measured experiments. The performance of the NLS–Transformer–LSTM model was thoroughly investigated. The results showed that the system can provide more accurate pseudo-DVL speed estimates. This capability ensures more stable and reliable underwater navigation accuracy during DVL failures. The authors believe that the proposed method provides powerful support for UAVs in challenging maritime environments.

Except for Velocity-Aided Navigation, Visual and Bio-Inspired Navigation is researched. An analysis and experimental validation of a vision-assisted inertial navigation algorithm for planetary landing applications are presented in [10]. The described system utilizes tight integration of inertial and vision measurements to compute accurate estimates of the lander’s relative position, orientation, and velocity in real time. Two types of features are considered. The first are imaged landmarks whose global 3D positions can be determined from a surface map. The second are opportunistic features that can be tracked in sequential images, but whose 3D positions are unknown. Both types of features are processed in an extended Kalman filter (EKF) estimator. These features are then optimally combined with IMU measurements. Touchdown test results for a sounding rocket showed error estimates of 0.16 m/s for velocity and 6.4 m for position. The obtained results allow for a significant improvement in the current state of the art of remote sensing navigation systems.

Unlike previous studies on AUV navigation, ref. [11] presents a new architecture for a ground vehicle navigation system. This vehicle is piloted remotely in conditions where GNSS signals are unstable and can be lost for long periods of time. The central feature of this system is an algorithm for recognizing environmental landmarks. This algorithm allows for continuous and accurate adjustment of the vehicle speed estimate. A sequential Kalman filter estimates the state and processes camera data to determine the vehicle’s position relative to the identified landmarks. A modern LiDAR serves as an odometer for landmark detection, allowing for low velocity determination errors. During testing, the system’s performance was evaluated on various real-world trajectories under various conditions. Test results showed that the system effectively determines speed when GNSS signals are distorted or absent.

Paper [12] developed “DeepLine-VIO,” which extracts illumination-invariant line features via an attraction-field-based deep network. This approach is far more robust than point-feature methods in low-texture environments, reducing Absolute Trajectory Error (ATE) by up to 15.87%. In [13], Visual Inertial Odometry (VIO) was implemented using on-sensor hardware acceleration for optical flow. By calculating motion vectors directly on the image sensor, they achieved a 49.4% reduction in latency and a 53.7% reduction in compute load, enabling 50 FPS operation on resource-constrained microcontrollers. Bio-inspired strategies are also maturing. Paper [14] integrated a polarization compass that mimics insect navigation. Using a chi-square test to detect magnetic anomalies, the system switches to the polarization heading reference in magnetic-denied environments. In [15], this was furthered by using SVM-based parameter tuning to classify sky conditions, ensuring accurate solar azimuth prediction under both clear and cloudy skies.

Paper [16] proposed a collaborative integrated navigation framework for UAV swarms that accounts for multiple uncertainties via statistical linearization, allowing drones to refine their positions by sharing relative ranging data. The papers [17,18] reviewed trajectory design paradigms and mutual localization techniques, highlighting that game-theory-based coalition formation and distributed consensus filtering are essential for maintaining swarm resilience without a central point of failure.

In addition to navigation, the following Velocity-Aided Attitude and Alignment of SINS are explored. In [19], the orientation of remotely piloted aircraft using measurements of gyroscopes, accelerometers, and GNSS signals is considered. Existing solutions provide limited stability guarantees. This is due to the following assumptions: local linearization, high-gain design, or the adoption of specific trajectories with constant vehicle acceleration. A new nonlinear observer for inertial orientation estimation is proposed. This allows for obtaining globally asymptotically and locally exponentially stable error dynamics [19]. The approach utilizes the mathematical apparatus of Lie group symmetry of the system dynamics to construct a globally admissible correction term. Simulation results showed that the estimation error converges to zero even under extremely unfavorable initial conditions.

Current trends in navigation system development point to the use of quantum sensors. Quantum sensors are expected to provide significant advantages for vehicle navigation [20]. This paper explores the potential of a hypothetical quantum IMU with significantly better performance than classical IMUs. The authors demonstrate that the significantly reduced noise level of accelerometers and gyroscopes cannot be automatically exploited. Before the start of the mission, it is proposed to perform the alignment phase with a reliable velocity sensor. Orientation errors are proposed to be estimated using numerical optimization. In this case, the estimate obtained by the inertial navigation method is compared with a reference velocity signal. Since quantum inertial measurement units (IMUs) provide much more accurate measurements, orientation errors can be compensated for much more accurately.

It should be noted that all of the mentioned works, except [20], deal with the issues of integrated SINS for the AUV. Our paper proposes the implementation of a VAN method using an IMU, without SINS, for drones and aircraft. The main results of this work are as follows:

(1) More complete expressions for SINS and VAN algorithm errors have been obtained.
(2) It is proposed to use the VAN algorithm at times when GNSS signals are not available.
(3) Semi-natural simulations confirmed the feasibility of VAN algorithms at times when GNSS signals are not available.

The paper consists of an introduction, four sections, and a conclusion. The main part of the paper derives an extended mathematical model of SINS error, which depends on the errors of gyroscopes and accelerometers. Then, based on the mathematical model of the new system and VAN algorithms, we derive a mathematical model of error, which depends on the errors of gyroscopes and the velocity meter. Using real-world data, we obtained graphical dependencies of the SINS errors and the new system, showing that the errors of the new system are several times smaller than those of an uncorrected SINS. Results of semi-natural experiments showed that the errors of the new system are at the level of those of a SINS integrated with GNSS. This work is based on simulated velocity input based on real trajectory data. The proposed method for determining latitude, longitude and altitude can be an alternative to the SINS, which is integrated with GNSS and can also serve as a backup navigation system.

In contrast to classical inertial navigation, where the translational velocity is determined by the integration of measured accelerations, in the proposed VAN architecture, the velocity is considered as a directly measured quantity by an independent sensor. As a result, the role of the MEMS IMU changes: gyroscopes provide orientation detection and conversion of the measured speed into a navigation coordinate system, while the determination of translational motion is based on direct velocity measurements. This results in a different structure for the accumulation of navigational errors compared to traditional SINSs.

Consequently, the proposed approach should be regarded not as a modification of the conventional strapdown inertial navigation algorithm, but as an alternative navigation architecture with a fundamentally different mechanism for determining translational motion.

## 2. Materials and Methods

### 2.1. Statement of the Problem

**Figure 1:**
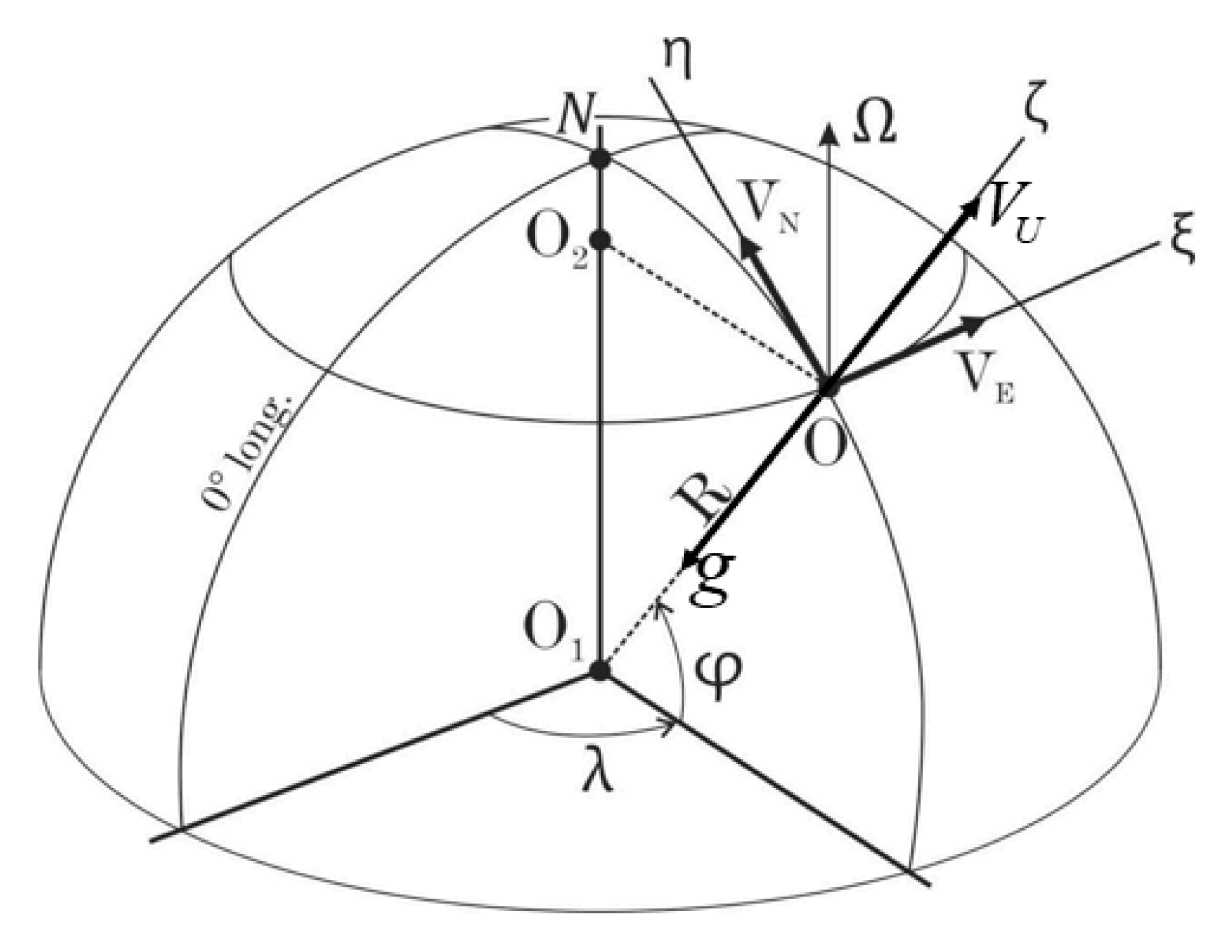
Illustration of the geographic reference system.

In Figure 1, a navigation reference frame Oξηζ is shown. Here, the axis Oξ is directed to the east, the axis Oη is directed to the north, and the axis Oζ is directed upwards (ENU).

The notations of variables used in this study are provided in Appendix A.

The spherical model of the Earth is accepted. The following notations are introduced here: λ is a longitude, φ is a latitude, *R* is a radius of the Earth; *h* is a flight altitude; Ω is an angular rate of the Earth’s rotation; *g* is gravity acceleration; $v_{N}=v\cos H;\;v_{E}=v\sin H;\;v_{U}$ are projections of the vehicle’s velocity *v* onto vertical axes Oζ; and *H* is a heading.

We can obtain the basic equations of inertial navigation [21] from Figure 1:

(1) $$\begin{array}{l} \dot{\varphi }=\frac{v_{N}}{R+h};\\ \dot{\lambda }=\frac{v_{E}}{\left(R+h\right)\cos\varphi };\\ \dot{h}=v_{U}. \end{array}$$

**Figure 2:**
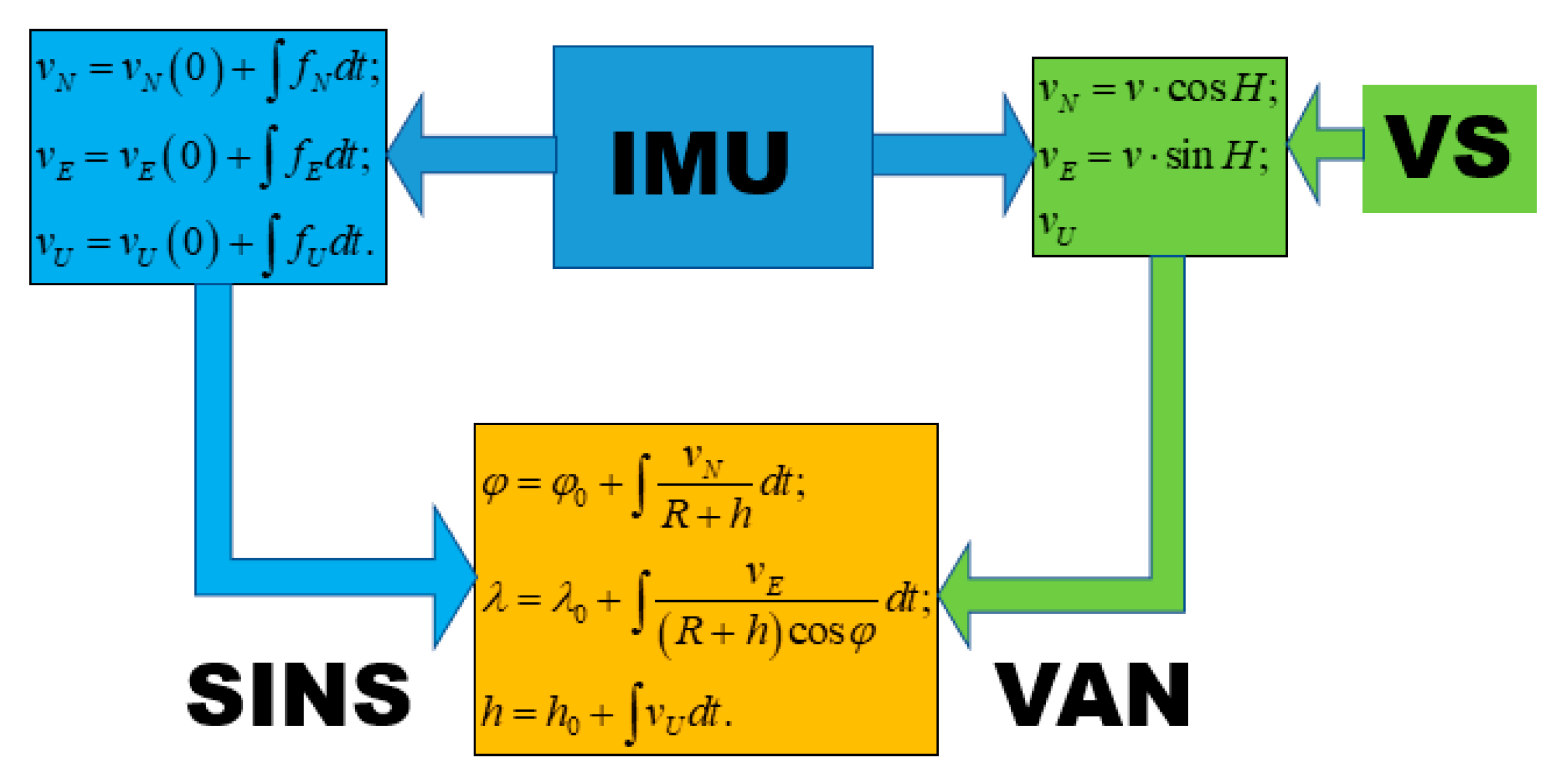
Two way, SINS and VAN of the navigation Equation (1) implementation.

By integrating Equation (1) over time, we can obtain the current values of latitude, longitude, and altitude. To do this, we need to have the velocity projections $v_{N},v_{E},v_{U}$. Velocity can be obtained, firstly, by using inertial SINS technology and integrating accelerometer signals (left side of Figure 2).

Second, another method of obtaining velocity is the so-called VAN method, in which velocities are obtained from velocity sensors (VSs), shown on the right side of Figure 2. As VSs, Doppler radar meters, an airborne signal system, Pitot tubes, and optical sensors such as Laser Velocity Sensors (LVSs) can be used in aviation [22]. Let us first consider the standard inertial method.

For both methods, SINS and VAN, the use of an IMU is necessary, which contains three gyroscopes and three accelerometers that are mutually orthogonal. The IMU is used to obtain a direction cosine matrix, $C_{b}^{n}$, which will be discussed below.

### 2.2. Inertial Method for Determining Velocity Projections

First, let us consider obtaining projections of the vehicle’s velocity $v_{N},v_{E},v_{U}$ using the inertial method or the standard method of classical SINS using accelerometers.

Derivatives of $v_{N},v_{E},v_{U}$ or projections of the vehicle’s acceleration onto the axes of the navigation reference frame can be obtained from the full expressions for the projections of the accelerometers’ specific forces $f_{N},f_{E},f_{U}$ and correctional signals $f_{N}^{c},f_{E}^{c},f_{U}^{c}$:

(2) $$\begin{array}{l} {\dot{v}}_{E}=f_{E}-f_{E}^{c};\\ {\dot{v}}_{N}=f_{N}-f_{N}^{c};\\ {\dot{v}}_{U}=f_{U}-f_{U}^{c}. \end{array}$$

Here $f_{N}^{c},f_{E}^{c},f_{U}^{c}$ are correctional signals that take into account portable movement:

(3) $$\begin{array}{l} f_{E}^{c}=\frac{v_{E}v_{U}}{R}-\frac{v_{N}v_{U}}{R}tg\varphi +2\left(v_{U}\Omega \cos\varphi -v_{N}\Omega \sin\varphi \right);\\ f_{N}^{c}=\frac{v_{N}v_{U}}{R}+\frac{v_{E}^{2}}{R}tg\varphi +2v_{E}\Omega \sin\varphi ;\\ f_{U}^{c}=\frac{v_{N}^{2}}{R}-\frac{v_{E}^{2}}{R}-2v_{E}\Omega \cos\varphi +g. \end{array}$$

**Figure 3:**
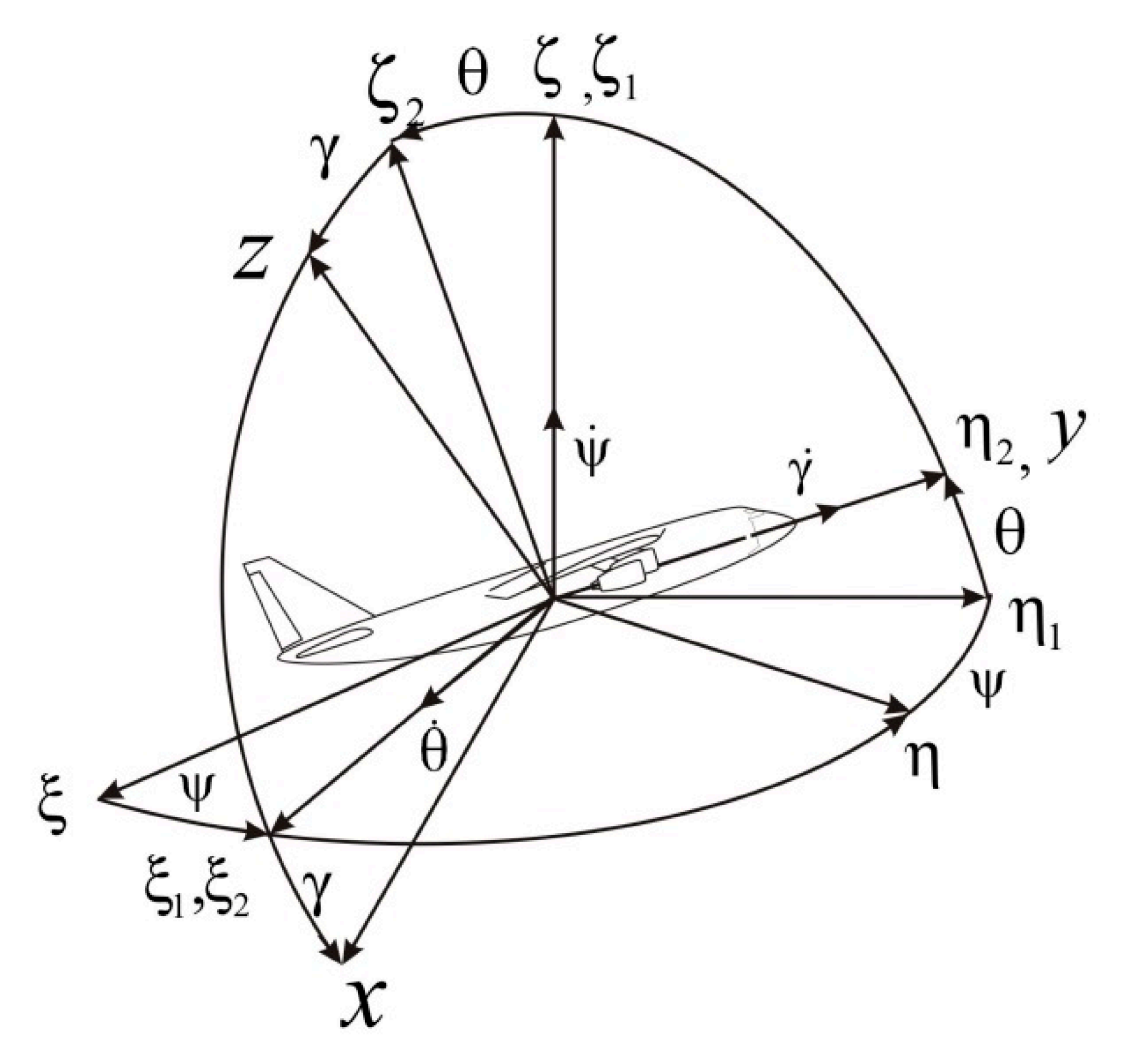
Definition of the vehicle rotation.

By integrating expressions (2), we get the desired velocity projections $v_{N},v_{E},v_{U}$. Since accelerometers are mounted on the vehicle, they measure accelerations relative to the axes associated with the vehicle, not the axes of the geographic reference frame. Let us establish a relationship between the navigation reference frame Oξηζ and the reference frame associated with the vehicle or body frame, *Oxyz*. Let us denote the vehicle’s rotation angles ψ,θ,γ (yaw, pitch, roll) relative to the navigation reference frame by Oξηζ (Figure 3).

For a given sequence of rotations, the direction cosine matrix $C_{n}^{b}$ of the transition from the reference frame Oξηζ to Oxyz will have the form

(4) $$C_{n}^{b}=\left[\begin{array}{ccc} \cos\gamma \cos\psi -\sin\gamma \sin\theta \sin\psi & \cos\gamma \sin\psi +\sin\gamma \sin\theta \cos\psi & -\sin\gamma \cos\theta \\ -\cos\theta \sin\psi & \cos\theta \cos\psi & \sin\theta \\ \sin\gamma \cos\psi +\cos\gamma \sin\theta \sin\psi & \sin\gamma \sin\psi -\cos\gamma \sin\theta \cos\psi & \cos\gamma \cos\theta \end{array}\right],$$

There is a relationship between the projections of the specific forces on the axes Oξηζ and Oxyz:

(5) $$\left[\begin{array}{c} f_{E}\\ f_{N}\\ f_{U} \end{array}\right]=C_{b}^{n}\left[\begin{array}{c} f_{x}\\ f_{y}\\ f_{z} \end{array}\right],$$

here, $C_{b}^{n}$ is a transposed matrix $C_{n}^{b}$ with elements

$$C_{b}^{n}=\left[\begin{array}{ccc} c_{11} & c_{12} & c_{13}\\ c_{21} & c_{22} & c_{23}\\ c_{31} & c_{32} & c_{33} \end{array}\right].$$

To find the matrix elements $C_{b}^{n}$, it is necessary to solve the orientation or vehicle attitude problem, i.e., determine the angles ψ,θ,γ. The orientation problem can be solved using Euler’s kinematic equations, generalized Poisson equations, quaternions, or the finite Euler rotation angle.

Now, knowing the orientation angles and therefore the matrix elements $C_{b}^{n}$, we can calculate the current latitude, longitude, and altitude using expressions (1), given the latitude, longitude, and altitude values from the previous step. For the first step of the calculations, we need to know the initial coordinates of the location.

### 2.3. Dependence of SINS Errors on the Errors of Accelerometers and Gyroscopes

From the fundamental equations of inertial navigation (1), by expansion into a Taylor series, we can obtain the biases of the SINS. In the future we will assume that *R* >> *h*. Omitting cumbersome transformations (details are available in Appendix B), we present expressions for the biases of the SINS:

(6) $$\begin{array}{l} \Delta \lambda =\frac{1}{R\cos\varphi }\left[\Delta f_{E}\frac{t^{2}}{2}+\left(f_{z}\cdot \Delta \omega_{y}-f_{y}\cdot \Delta \omega_{z}\right)\frac{t^{3}}{6}+\left(f_{y}\cdot \omega_{x}\cdot \Delta \omega_{y}-f_{z}\cdot \omega_{z}\cdot \Delta \omega_{x}\right)\frac{t^{4}}{24}+\ldots \right];\\ \Delta \varphi =\frac{1}{R}\left[\Delta f_{N}\frac{t^{2}}{2}+\left(f_{x}\cdot \Delta \omega_{z}-f_{z}\cdot \Delta \omega_{x}\right)\frac{t^{3}}{6}-\left(f_{x}\cdot \omega_{x}\cdot \Delta \omega_{y}+f_{z}\cdot \omega_{z}\cdot \Delta \omega_{y}\right)\frac{t^{4}}{24}+\ldots \right];\\ \Delta h=\Delta f_{U}\frac{t^{2}}{2}+\left(f_{y}\cdot \Delta \omega_{x}-f_{x}\cdot \Delta \omega_{y}\right)\frac{t^{3}}{6}+\left(f_{x}\cdot \omega_{z}\cdot \Delta \omega_{x}+f_{y}\cdot \omega_{z}\cdot \Delta \omega_{y}\right)\frac{t^{4}}{24}+\ldots , \end{array}$$

where Δλ is a longitude determination error; Δφ is a latitude determination error; Δ*h* is an altitude determination error; $\Delta \omega_{x},\Delta \omega_{y},\Delta \omega_{z}$ are biases or drifts of gyroscopes; $\omega_{x},\omega_{y},\omega_{z}$ are the vehicle’s angular velocity projections onto the vehicle reference frame, measured by three gyroscopes; and *t* is a time. Here $\Delta f_{E},\Delta f_{N},\Delta f_{U}$ are the biases of accelerometer projections onto the navigation reference frame and $\Delta f_{x},\Delta f_{y},\Delta f_{z}$ are the biases of accelerometer projections onto the vehicle’s reference frame. They are related by the relation (5) too.

It is evident that the errors of the SINS, except for the first term in (6), depend on the actual values of specific forces $f_{x},f_{y},f_{z}$ and the vehicle’s angular rate $\omega_{x},\omega_{y},\omega_{z}$. From expressions (6) in coordinate form follows the vector form (7) for linear errors:

(7) $$e=\Delta f\cdot \frac{t^{2}}{2!}+\left(\Delta \omega \times f\right)\cdot \frac{t^{3}}{3!}+\left(\Delta \omega \times \omega -b\right)\times f\cdot \frac{t^{4}}{4!}+\ldots ,$$

where $e\left(\Delta \lambda \cdot R\cos\varphi ,\Delta \varphi \cdot R,\Delta h\right)$ is a vector of linear systematic error of SINS; $\Delta f\left(\Delta f_{E},\Delta f_{N},\Delta f_{U}\right)$ is an error vector of accelerometers; $\Delta \omega \left(\Delta \omega_{x},\Delta \omega_{y},\Delta \omega_{z}\right)$ is a gyroscope error vector; $f\left(f_{x},f_{y},f_{z}\right)$ is a vector of the specific forces; and $b\left(-\Delta \omega_{z}\omega_{y},\Delta \omega_{y}\omega_{x},\Delta \omega_{x}\omega_{y}\right)$. The symbol ‘x’ denotes the vector product of two vectors.

In addition to systematic components, gyroscopes and accelerometers are characterized by random errors of the Angular Random Walk (ARW) [deg/$\sqrt{hr}$] and Velocity Random Walk (VRW) [m/s/$\sqrt{hr}$] types, respectively. It can be shown (details are available in Appendix B) that these errors lead to standard deviation errors of the SINS:

(8) $$\sigma_{*}=VRW\cdot \frac{t^{\frac{3}{2}}}{\sqrt{3}}+\left(ARW\times f\right)\cdot \frac{t^{\frac{5}{2}}}{2\sqrt{5}}+\left(ARW\times \omega -b\right)\times f\cdot \frac{t^{\frac{7}{2}}}{6\sqrt{7}}+\ldots .$$

Here $VRW\left(VRM_{E},VRM_{N},VRM_{U}\right)$ is a vector of the VRW; $ARW\left(ARW_{x},ARW_{y},ARW_{z}\right)$ is a vector of the ARW.

An analysis of the SINS error expressions (7) and (8) reveals a dramatic time dependence. To address this drawback, various types of SINS corrections are used from navigation systems built on other physical principles. The most common of such navigation systems currently are GNSS satellite navigation systems. However, integrating SINS with GNSS results in the integrated system’s dependence on the operation of the satellite navigation system, which can be unstable in adverse conditions, such as urban canyons in modern cities, signal loss from satellites, etc. In this case, an alternative method based on Velocity-Aided Navigation using MEMS IMU gyroscopes is proposed.

### 2.4. Determining Latitude, Longitude, and Altitude Using Velocity-Aided Navigation

Velocity-Aided Navigation is based on Equation (1) too. While accelerometers are used to determine the object’s velocity for SINS, Velocity-Aided Navigation uses Doppler radar, Pitot tubes, and optical sensors such as Laser Velocity Meters (LVMs) in aviation. A Doppler velocity log (DVL) is used at sea, and a Wheel Odometer with an encoder is used for land vehicles. Despite their different operating principles, they all share the commonality of measuring relative velocity.

By integrating expressions (1), we obtain the current latitude, longitude, and altitude values, knowing the object’s current velocity and heading, as well as the latitude, longitude, and altitude values from the previous step. For the first step of the calculation, we need to know the initial coordinates of the location.

From Equation (1), by expanding them into a Taylor series, we obtain the biases of VAN, which depend on the biases of the velocity sensors and gyroscopes:

(9) $$\begin{array}{l} \Delta \lambda =-\frac{1}{R\cos\varphi }\left[\sin\psi \cdot \Delta v\cdot t+V_{N}\left(\Delta \omega_{z}\frac{t^{2}}{2}-\omega_{x}\cdot \Delta \omega_{y}\frac{t^{3}}{6}\right)+\ldots \right];\\ \Delta \varphi =\frac{1}{R}\left[\cos\psi \cdot \Delta v\cdot t+V_{E}\left(\Delta \omega_{z}\frac{t^{2}}{2}-\omega_{x}\cdot \Delta \omega_{y}\frac{t^{3}}{6}\right)+\ldots \right];\\ \Delta h=\Delta V_{U}\cdot t+\ldots , \end{array}$$

where Δ*v* and Δ*V_U_* are the biases of horizontal and vertical velocity measurements, and *t* is time.

It is obvious that the errors of the VAN system, except for the first term in (9), depend on the actual values of the linear velocity and angular velocity of the object.

In addition to systematic components, horizontal and vertical velocity measurements are characterized by random errors of the Position Random Walk (PRW) type [m/$\sqrt{h}$]. It can be shown that these errors lead to standard deviation errors of the VAN/IMU:

(10) $$\begin{array}{l} \sigma_{\lambda }=-\frac{1}{R\cos\varphi }\left[\sin\psi \cdot PRW\cdot \sqrt{t}+V_{N}\left(ARW_{z}\frac{t^{\frac{3}{2}}}{\sqrt{3}}-\omega_{x}\cdot ARW_{y}\frac{t^{\frac{5}{2}}}{2\sqrt{5}}\right)+\ldots \right];\\ \sigma_{\varphi }=\frac{1}{R}\left[\cos\psi \cdot PRW\cdot \sqrt{t}+V_{E}\left(ARW_{z}\frac{t^{\frac{3}{2}}}{\sqrt{3}}-\omega_{x}\cdot ARW_{y}\frac{t^{\frac{5}{2}}}{2\sqrt{5}}\right)+\ldots \right];\\ \sigma_{z}=PRW_{z}\cdot \sqrt{t}+\ldots . \end{array}$$

Here, *PRW* and *PRW*_z_ are the random errors of velocity sensors; *ARW_y_* and *ARW_z_* are the random drifts of the gyroscopes.

Table 1 presents the numerical characteristics of the errors of MEMS gyroscopes and accelerometers (data of Inertial Labs INS-P^®^ is placed in Supplementary Materials), as well as velocity meters.

**Figure 4:**
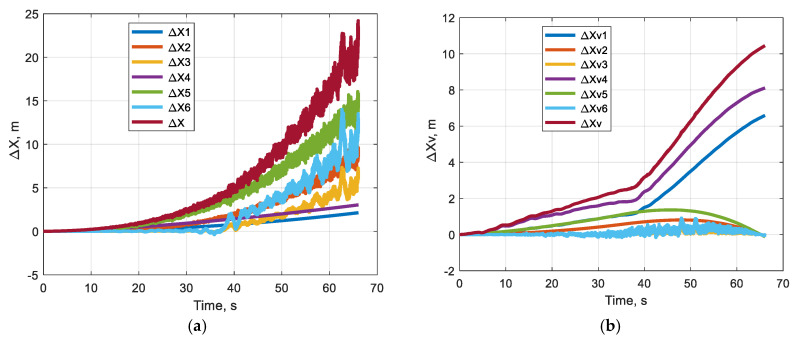
SINS (**a**) and VAN (**b**) errors for Δ*x* and Δ*x_v_*.

**Figure 5:**
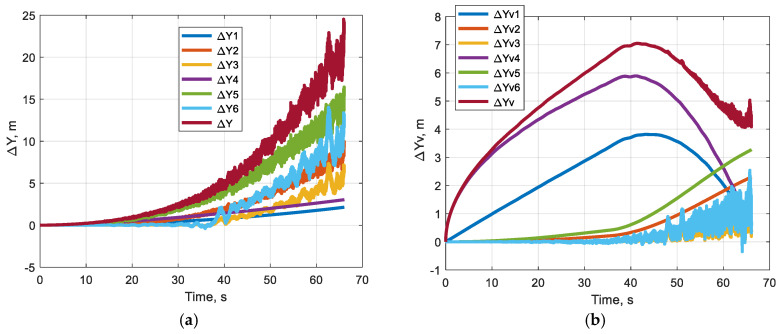
SINS (**a**) and VAN (**b**) errors for Δ*y* and Δ*y_v_*.

**Figure 6:**
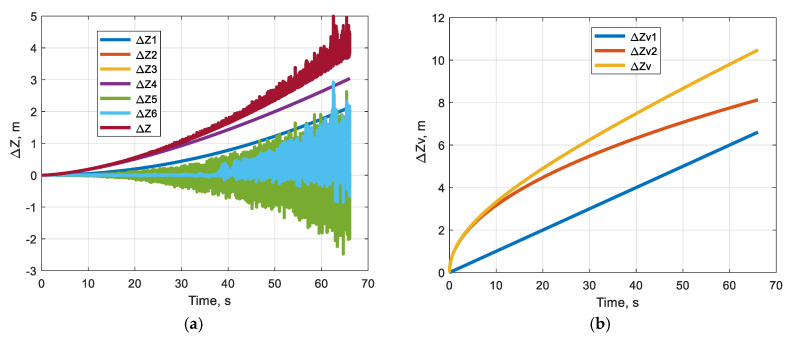
SINS (**a**) and VAN (**b**) errors for Δ*z* and Δ*z_v_*.

Figure 4, Figure 5 and Figure 6 show the calculation results for the errors SINS (a) and VAN (b) for 65 s in the following form:

(11) $$\Delta e=\sqrt{\sum_{i}\Delta e_{i}^{2}},$$

where Δe and $\Delta e_{i}$ are the total errors and their components respectively; *e = p*, *q*; *p* = *x*, *y*, *z* (SINS); *q* = *x_v_*, *y_v_*, *z_v_* (VAN); and Δx=Δλ⋅Rcosφ; Δy=Δφ⋅R; Δz=Δh; all expressions for $\Delta e_{i}$ are placed in Appendix A.

Also, the actual values of the vehicle’s acceleration, linear velocity, and angular velocity from the flight data were used in the calculations (11).

As can be seen from the figures, the errors of the VAN method for the *X* and *Y* coordinates over a period of 65 s are significantly less than those of the inertial method.

## 3. Results of Semi-Natural Experiment of VAN and SINS/GNSS Operation

**Figure 7:**
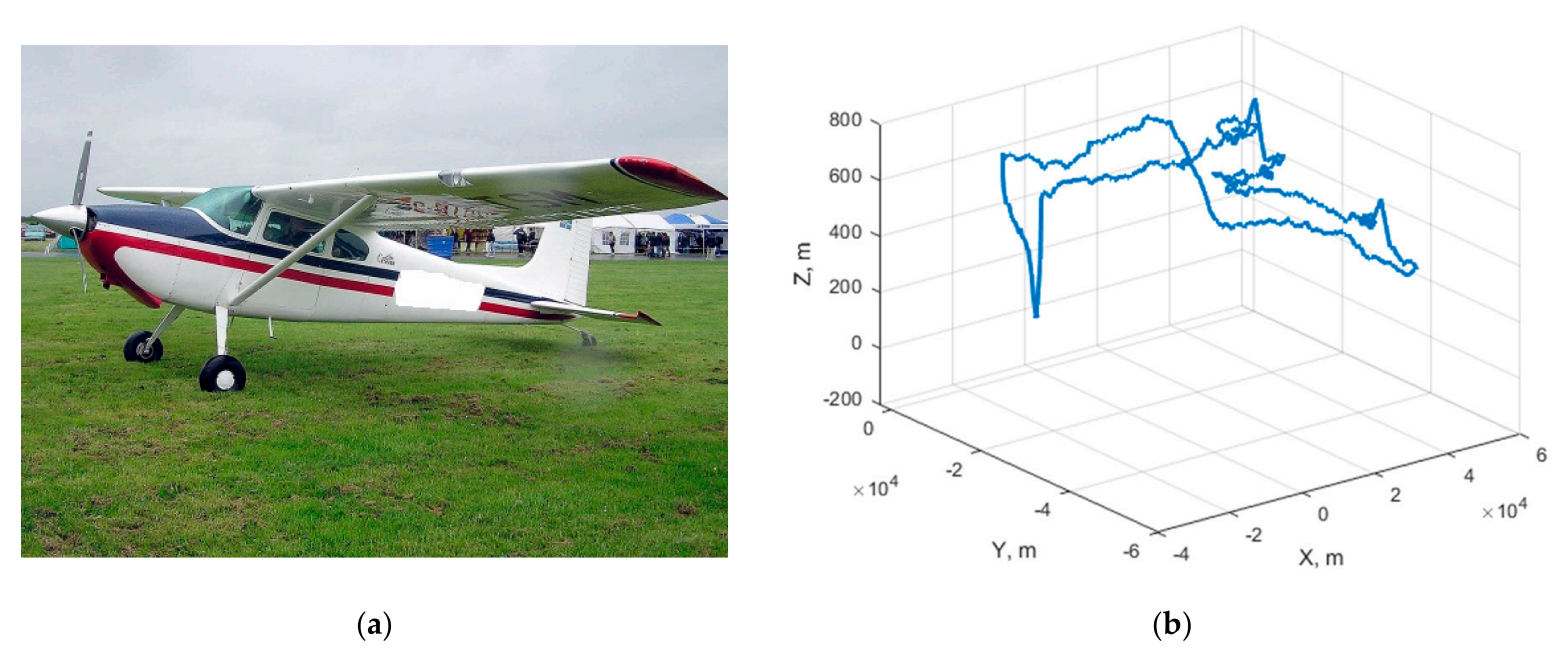
The Cessna aircraft (**a**) and the flight trajectory with a duration of 104 min (**b**).

To test the effectiveness of the developed method for autonomously determining latitude and longitude, SINS data obtained during a flight on a small Cessna aircraft (Figure 7a) near Orlando, FL, USA, was used, and the VAN algorithm was simulated. Figure 7b shows the flight trajectory with a duration of 104 min. Inertial Labs INS-P^®^ with connected GNSS antenna Tallysman TW3972^®^ was used. The link for the data of Inertial Labs INS-P^®^ and Tallysman TW3972^®^ are placed in the Supplementary Materials at the end of our article. After SINS installation on the aircraft, the 2D calibration of the SINS magnetometer was performed using Inertial Labs GUI^®^ software with a 0.3 degree of heading error. Test conditions provided a clear sky for the GNSS antenna, so the SINS received GNSS data without loss. The SINS data included information on the aircraft’s heading, pitch, and roll angles; angular velocity; specific forces; latitude, longitude, and altitude; north, east, and vertical velocity; and the current time. Also, the SINS data included position and velocity data provided by an embedded GNSS receiver. The GNSS position was used as a reference for the evaluation of VAN performance.

The initialization procedure for VAN is the same as for MEMS SINS: IMU accelerometers are used for initial alignment and a magnetometer is used for initial heading calculation. Initial latitude, longitude, and altitude are external data. At the beginning, the system operates as a conventional SINS; then, in the GNSS denied environment, the system algorithm is switched to VAN instead of SINS.

In the proof-of-concept phase, GNSS is used as a high-precision reference speed source that allows evaluation of the proposed VAN architecture while minimizing the influence of imperfections associated with a particular velocity sensor. The GNSS-derived velocity used in the experiments serves exclusively for validation purposes as a convenient surrogate for an independent velocity sensor, because an actual onboard velocity sensor was unavailable during flight tests. The proposed navigation algorithm itself is independent of GNSS and requires only velocity measurements, which may originate from Pitot tubes, Doppler velocity logs, wheel encoders, optical sensors, or other dedicated velocity sensors.

**Figure 8:**
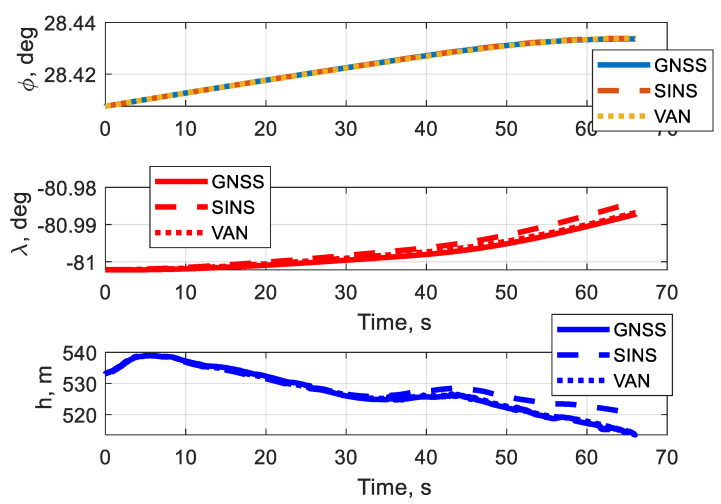
Values of latitude, longitude and altitude for the first leg of the flight.

**Figure 9:**
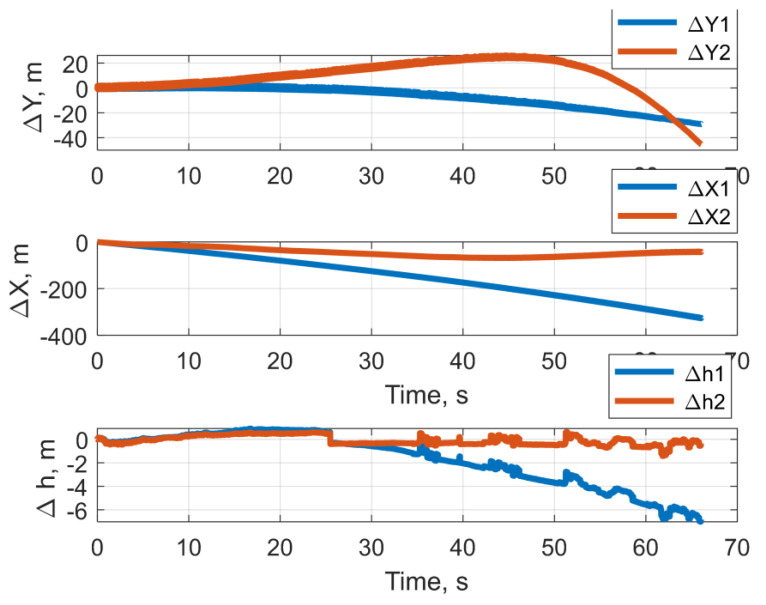
Errors in determining linear coordinates for the first leg of the flight.

**Figure 10:**
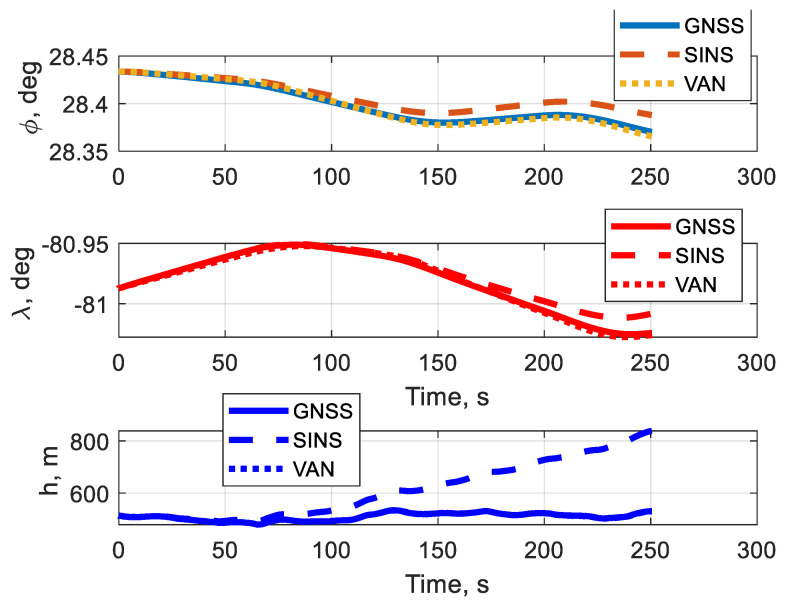
Values of latitude, longitude and altitude for the second leg of the flight.

**Figure 11:**
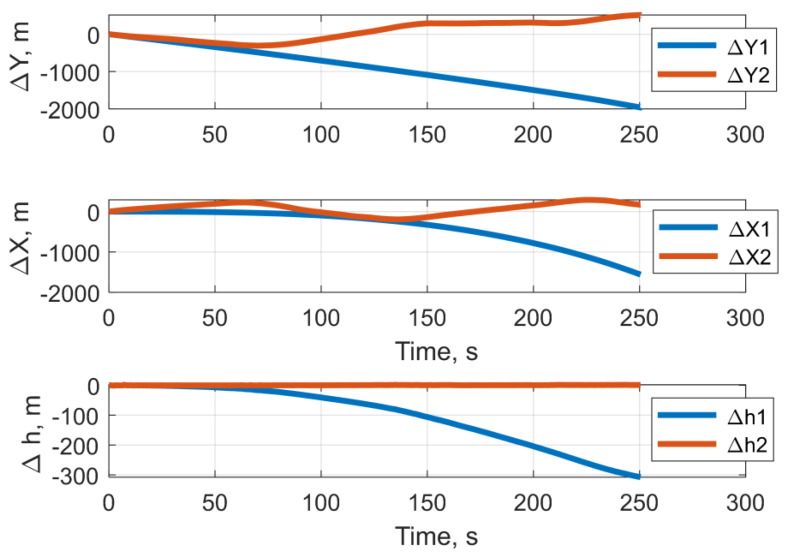
Errors in determining the linear coordinates for the second leg of the flight.

**Figure 12:**
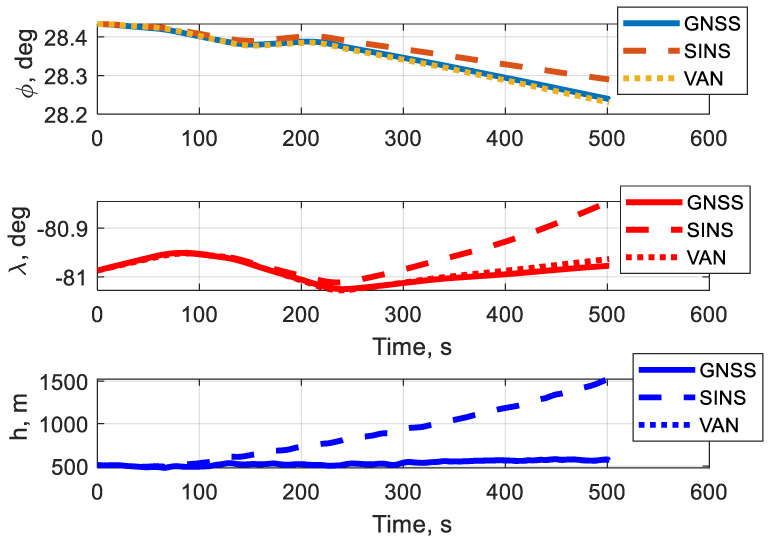
Values of latitude, longitude, and altitude for the third leg of the flight.

**Figure 13:**
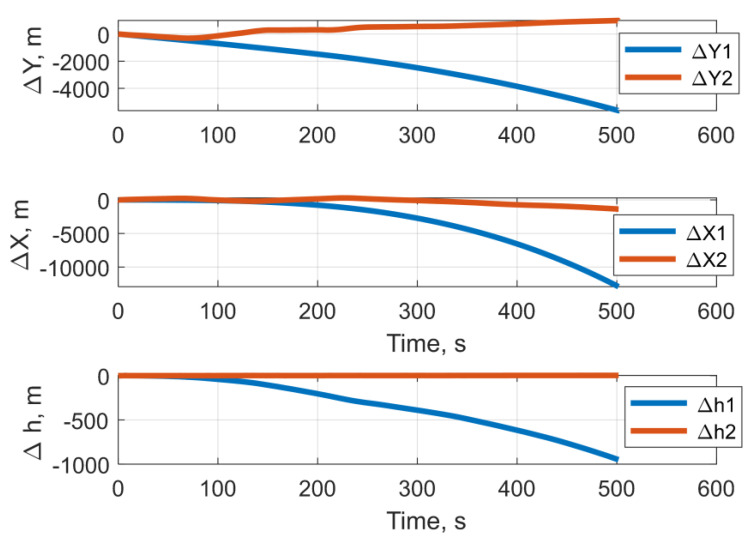
Errors in determining the linear coordinates for the third leg of the flight.

Figure 8, Figure 9, Figure 10, Figure 11, Figure 12 and Figure 13 show the graphical dependences of latitude, longitude, and altitude over time, and the error in determining linear coordinates over time for three flight segments. The following notations are used in the figures: the GNSS curve represents the latitude, longitude, and altitude values measured using a GNSS receiver; the SINS curve represents the latitude, longitude, and altitude values calculated using the standard SINS algorithms; and the VAN curve represents the latitude, longitude, and altitude values calculated using the proposed VAN algorithms. The results of the experimental testing of the operation of VAN and SINS for the first leg of the flight lasting 65 s are shown in Figure 8 and Figure 9.

From Figure 8 it can be seen that the latitude, longitude and altitude angles measured using the GNSS receiver, the latitude, longitude, and altitude values calculated using the standard SINS algorithms and the latitude, longitude, and altitude values calculated using the proposed VAN algorithms are almost identical.

In Figure 9, the curves Δ*q_i_* (*q = x*, *y*, *h*; *i =* 1, 2) represents the error of the SINS and the VAN and between GNSS information:

(12) $$\begin{array}{l} \Delta x_{1}=\left(\lambda_{SINS}-\lambda_{GNSS}\right)\cdot R\cos\varphi ;\;\\ \Delta y_{1}=\left(\varphi_{SINS}-\varphi_{GNSS}\right)\cdot R;\;\\ \Delta h_{1}=h_{SINS}-h_{GNSS}; \end{array}\;\begin{array}{l} \Delta x_{2}=\left(\lambda_{VAN}-\lambda_{GNSS}\right)\cdot R\cos\varphi ;\;\\ \Delta y_{2}=\left(\varphi_{VAN}-\varphi_{GNSS}\right)\cdot R;\;\\ \Delta h_{2}=h_{VAN}-h_{GNSS}. \end{array}$$

Table 2 presents the mean values, maximum and minimum values, and unbiased root mean square error (RMSE) and mean absolute error (MAE) for the errors (12) that were calculated for the first leg of the flight. The MAE and RMSE have been calculated using Equations (13) and (14):

(13) $$MAE_{j}=\frac{1}{N}\left|\Delta x_{ji}\right|,\;j=1,2;\;i=1,2,\ldots ,N;$$

(14) $$RMSE_{j}=\sqrt{\frac{1}{N-1}\sum_{i=1}^{N}{\left(\Delta x_{ji}\right)}^{2}}.$$

From Figure 8 and Table 2, it can be concluded that SINS errors are significantly higher than VAN errors, except coordinate *y*. Thus, for example, the ratio of MAE values for the three linear coordinates Δ*x_j_*, Δ*y_j_*, and Δ*h_j_* (*j* = 1, 2) is 3.4, 0.56 and 5.62, respectively.

The results of the experimental testing of the operation of VAN and SINS for the second leg of the flight lasting 250 s are shown in Figure 10 and Figure 11.

From Figure 10, it can be seen that the latitude, longitude, and altitude angles measured using the GNSS receiver, the latitude, longitude, and altitude values calculated using the SINS algorithms and the latitude, longitude, and altitude values calculated using the proposed VAN algorithms are different, especially for SINS.

Table 3 presents the mean values, maximum and minimum values, and unbiased RMSE and MAE for the errors (12) that were calculated for the second leg of the flight.

From Figure 11 and Table 3, we can see that SINS errors are significantly higher than VAN errors, especially for altitude. Thus, for example, the ratio of MAE values for linear coordinates Δ*x_j_*, Δ*y_j_*, and Δ*h_j_* (*j* = 1, 2) is 2.73, 3.88 and 232, respectively.

The results of the experimental verification of the VAN and SINS systems for the third segment, lasting 500 s, are shown in Figure 12 and Figure 13.

From Figure 12 it can be seen that the latitude, longitude and altitude values measured using the GNSS receiver, and the latitude, longitude and altitude values calculated using the proposed VAN algorithms are almost identical. However, the latitude, longitude and altitude values calculated using the SINS algorithms are very different compared to GNSS and VAN.

Table 4 presents the mean values, maximum and minimum values, and unbiased RMSE and MAE for the errors (12) that were calculated for the third flight segment.

From Figure 13 and Table 4, it can be concluded that the that SINS errors are significantly higher than VAN errors, especially for altitude. Thus, for example, the ratio of MAE values for the three linear coordinates Δ*x_j_*, Δ*y_j_*, and Δ*z_j_* (*j* = 1, 2) is 9.03, 4.73 and 328, respectively. The magnitude of the obtained errors indicates high errors for the proposed method for 8 min of the flight without outer correction. Thus, the results shown in Figure 9, Figure 11 and Figure 13, as well as the data from Table 2, Table 3 and Table 4, fully confirm the results of theoretical studies given in the first part of our manuscript and the obtained expressions (6)–(8) for standard SINS, and (9) and (10) for the VAN method.

**Figure 14:**
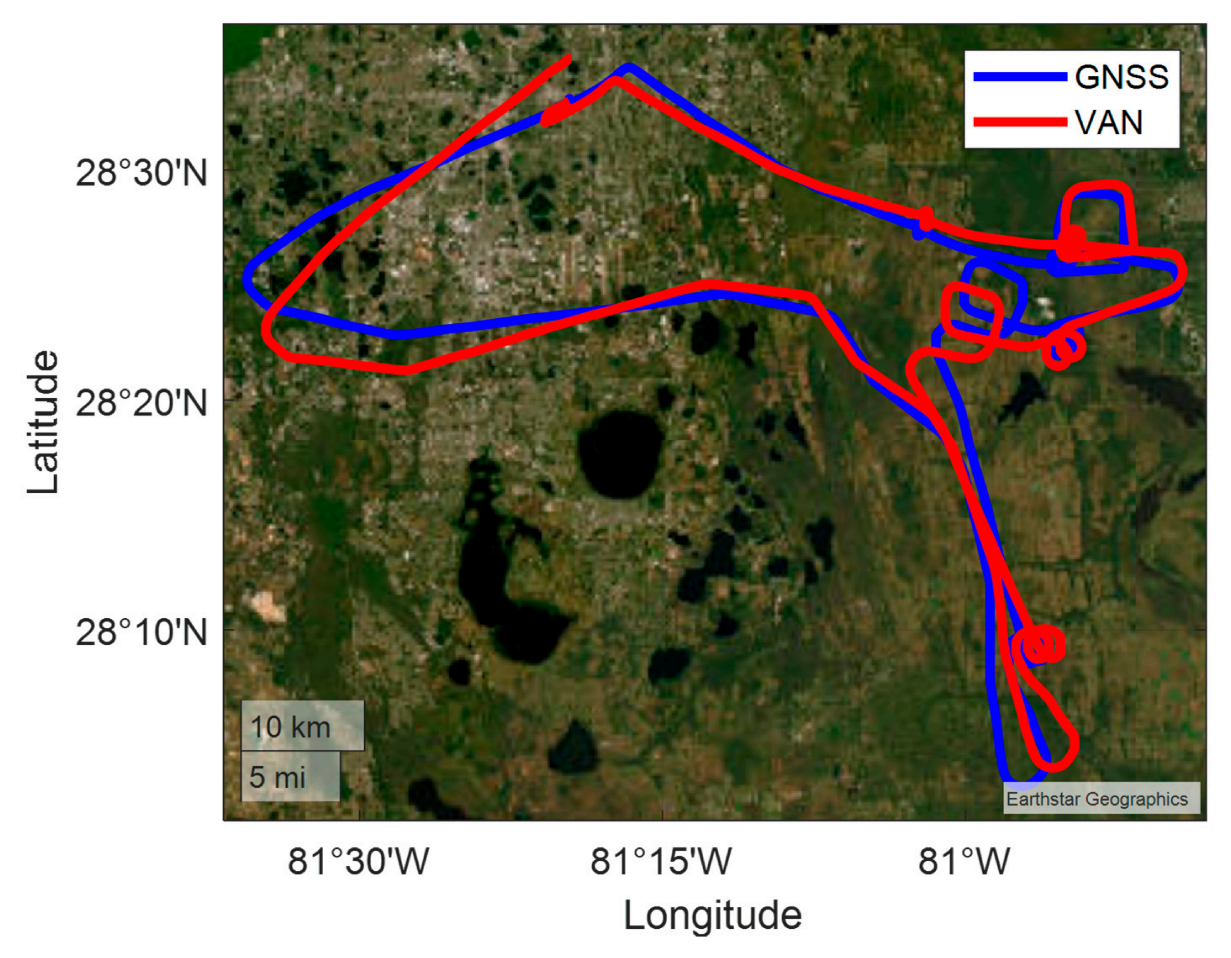
The trajectory curves for the total flight time of 104 min.

The calculated trajectory curves for the total flight time of 104 min are shown in Figure 14. The blue curve is plotted for GNSS data. The red curve is obtained from the computing of the VAN algorithms. Within 104 min, the MAE of the VAN method reached some kilometers for *x* and *y* coordinates respectively. It should be noted that VAN worked autonomously without GNSS correction. However, as previous calculations have shown, on short intervals, the errors of the VAN method are quite acceptable. This indicates that the VAN method can be used as an alternative to SINS/GNSS, in conditions where GNSS signals are not available.

## 4. Discussion

Today, GNSS is definitely the leader among navigation systems due to its advantages. Unfortunately, GNSSs are not autonomous systems. At the same time, SINSs are autonomous, but their errors, as shown above, increase greatly over time according to expressions (7) and (8). Therefore, in practice, SINS is integrated with GNSS. However, such integrated SINS/GNSSs become non-autonomous. We have considered the possibility of using the VAN method to create an autonomous system.

As semi-natural experiments have shown, the proposed VAN method may well be an alternative to standard SINS/GNSS methods in GNSS-denied environments. This will be especially useful for use for movement intervals of up to 10 min. The ratio of the MAE values for three linear coordinates Δ*x_j_*, Δ*y_j_*, and Δ*z_j_* (*j* = 1, 2) are 3.4, 0.56 and 5.62, respectively, for the first part of the path within 65 s; 2.73, 3.88 and 232 for the second part within 250 s; and 9.03, 4.73 and 328 within 500 s. The authors were forced to conduct a semi-natural experiment, since there were no modern speed sensors, and air signal receivers (Pitot tubes) gave significant errors in the presence of strong winds. In the future, it is planned to conduct an experimental test of the VAN method using LVM or other Doppler velocity sensors. In this experiment, we used algorithmic compensation for errors according to expressions (9) and (10). In addition, the algorithmic compensation of errors of the VAN method for a long time, as well as integration with other navigation systems, is obviously a promising direction. One method to improve the accuracy of the VAN method for long periods of no GNSS signal can be to use Visual algorithms to integrate SINS/VAN/Visual.

## 5. Conclusions

The use of VAN is proposed as an alternative method for determining navigation parameters such as latitude, longitude, and altitude in GNSS-denied environments. Extended analytical dependencies of systematic and random errors over time are obtained for the standard SINS and the alternative method. Error calculations under real-world operating conditions show that the errors of the new method are significantly lower than those of the SINS. To experimentally test a new method for autonomously determining latitude and longitude, experimental flight data from a small Cessna aircraft were used. This data included the output signals of three MEMS gyroscopes, three MEMS accelerometers, latitude, longitude, and altitude information measured by a GNSS receiver, as well as horizontal and vertical velocity data and the current time. Based on flight data, attitude angles, current latitude, longitude, and altitude, as well as linear coordinate determination errors, calculations were performed. In addition, current latitude, longitude, and altitude values were calculated using SINS algorithms. The results of calculations based on experimental data demonstrate the effectiveness of the proposed method. Thus, for the first flight segment, which lasted 65 s, the errors in determining the linear coordinates of the alternative VAN methods were several times less than the SINS errors. For the other two flight segments, lasting 250 s and 500 s, the errors in determining the linear coordinates of the alternative VAN method were an order of magnitude less than the SINS errors. Thus, the proposed VAN method for determining navigation parameters using vehicle velocity can be used as an alternative for aircraft when GNSS signals are unavailable. The semi-natural experiment was intentionally designed to separate the validation of the proposed navigation architecture from the performance evaluation of a specific velocity sensor technology.

## Figures and Tables

**Table 1:** Performance parameters of the MEMS gyroscopes and accelerometers, as well as velocity meters.

Errors Description	Units	Value
Systematic bias of accelerometers	g	1 × 10^−4^
Velocity Random Walk (VRW)	$m/s/\sqrt{h}$	0.015
Systematic bias of gyroscopes	deg/s	2 × 10^−3^
Angular Random Walk (ARW)	$\deg/\sqrt{h}$	0.2
Systematic bias of the velocity sensor	m/s	1.0
Position Random Walk (PRW)	$m/\sqrt{h}$	60

**Table 2:** Calculated statistical estimates for the first flight segment.

	Δ*x*_1_, *m*	Δ*x*_2_, *m*	Δ*y*_1_, *m*	Δ*y*_2_, *m*	Δ*h*_1_, *m*	Δ*h*_2_, *m*
Mean	−147.38	−43.722	−7.4161	9.0020	−1.6709	−0.0932
Max	0	0	2.5058	26.182	0.9178	0.6211
Min	−327.36	−68.819	−29.150	−45.446	−7.0217	−1.4256
MAE	147.38	43.722	7.9477	13.966	2.0218	0.3592
RMSE	93.728	19.692	8.5394	9.3854	1.9386	0.1886

**Table 3:** Calculated statistical estimates for the second flight segment.

	Δ*x*_1_, *m*	Δ*x*_2_, *m*	Δ*y*_1_, *m*	Δ*y*_2_, *m*	Δ*h*_1_, *m*	Δ*h*_2_, *m*
Mean	−383.85	75.579	−921.75	79.643	−101.47	0.2477
Max	2.6878	291.91	0	514.55	1.5075	1.7771
Min	−1561.5	−193.59	−1964.0	−307.05	−307.67	−1.2166
MAE	384.07	140.73	921.75	237.02	101.48	0.4381
RMSE	439.06	78.383	559.23	119.18	97.335	0.3341

**Table 4:** Calculated statistical estimates for the third flight segment.

	Δ*x*_1_, *m*	Δ*x*_2_, *m*	Δ*y*_1_, *m*	Δ*y*_2_, *m*	Δ*h*_1_, *m*	Δ*h*_2_, *m*
Mean	−3216.1	−238.91	−2254.6	397.64	−340.39	0.9451
Max	2.1464	291.39	0	998.65	1.4757	2.4304
Min	−12,923	−1362.3	−5638.9	−307.12	−949.97	−1.2086
MAE	3216.2	356.07	2254.6	476.24	340.39	1.0371
RMSE	3673.9	364.72	1586.0	276.59	280.13	0.7340

## Data Availability

The original contributions presented in this study are included in the article/Supplementary Material. Further inquiries can be directed to the corresponding author.

## Supplementary Materials

The following supporting information can be downloaded at https://inertiallabs.com/wp-content/uploads/2025/12/INS-P_INS-P-OEM_Datasheet_REV1.00_DEC2025.pdf, https://www.navtechgps.com/tw3972_triple_band_gnss_antenna/.

## Abbreviations

The following abbreviations are used in this manuscript:

VAN	Velocity-Aided Navigation
VIO	Visual Inertial Odometry
GNSS	Global Navigation Satellite System
INS	Inertial Navigation System
SINS	Strapdown Inertial Navigation System
IMU	Inertial Measurement Unit
MEMS	Micro Electro Mechanical System
ARW	Angular Random Walk
VRW	Velocity Random Walk
PRW	Position Random Walk
VS	Velocity Sensor
DVL	Doppler Velocity Log
LVM	Laser Velocity Meter
AUV	Autonomous Underwater Vehicle
PS	Pressure Sensor
EKF	Extended Kalman Filter
RMSE	Root Mean Square Error
MAE	Mean Absolute Error

## Appendix A

Notations and the reference frames.

**Symbols**	**Description**
*Oξης*	The navigation reference frame (ENU), Figure 1
*Oxyz*	The reference frame associated with the vehicle
$v_{N},v_{E},v_{\varsigma }$	The projections of the vehicle’s velocity
φ, λ, *h*	The latitude, longitude and altitude
*R*, Ω	Earth’s radius and angular rate
$f_{E},f_{N},f_{U}$	The projections of the vehicle’s specific forces on to *Oξης*
$f_{x},f_{y},f_{z}$	The projections of the vehicle’s specific forces on to *Oxyz*
ψ,θ,γ	Yaw, pitch, roll angles, Figure 2
*H*	Heading
$C_{n}^{b}$	The direction cosine matrix of the transition from the reference frame Oξηζ to Oxyz
$C_{b}^{n}$	$\mathrm{The}\;\mathrm{transposed}\;\mathrm{matrix}\;C_{n}^{b}$
$c_{11},c_{12},c_{13},\ldots$	$\mathrm{Elements}\;\mathrm{of}\;\mathrm{the}\;\mathrm{direction}\;\cos\mathrm{ine}\;\mathrm{matrix}\;C_{b}^{n}$
$\omega_{x},\omega_{y},\omega_{z}$	The projections of the vehicle’s angular rate on to *Oxyz*
Δφ, Δλ, Δ*h*	Errors of the latitude, longitude and altitude determination
$\Delta f_{E},\Delta f_{N},\Delta f_{U}$	The projections of the biases of accelerometers on to *Oξης*
$\Delta f_{x},\Delta f_{y},\Delta f_{z}$	The biases of accelerometers
$\Delta \omega_{x},\Delta \omega_{y},\Delta \omega_{z}$	The biases or drifts of gyroscopes
Δx,Δy,Δz	The projections of the SINS linear total errors
$\Delta x_{1},\Delta y_{1},\Delta z_{1}$	$\Delta x_{1}=\Delta f_{E}\frac{t^{2}}{2},$$\;\Delta y_{1}=\Delta f_{N}\frac{t^{2}}{2},$$\;\Delta z_{1}=\Delta f_{U}\frac{t^{2}}{2}$
$\Delta x_{2}$	$\Delta x_{2}=\left(f_{z}\cdot \Delta \omega_{y}-f_{y}\cdot \Delta \omega_{z}\right)\frac{t^{3}}{6}$
$\Delta y_{2}$	$\Delta y_{2}=\left(f_{x}\cdot \Delta \omega_{z}-f_{z}\cdot \Delta \omega_{x}\right)\frac{t^{3}}{6}$
$\Delta z_{2}$	$\Delta z_{2}=\left(f_{y}\cdot \Delta \omega_{x}-f_{x}\cdot \Delta \omega_{y}\right)\frac{t^{3}}{6}$
$\Delta x_{3}$	$\Delta x_{3}=\left(f_{y}\cdot \omega_{x}\cdot \Delta \omega_{y}-f_{z}\cdot \omega_{z}\cdot \Delta \omega_{x}\right)\frac{t^{4}}{24}$
$\Delta y_{3}$	$\Delta y_{3}=-\left(f_{x}\cdot \omega_{x}\cdot \Delta \omega_{y}+f_{z}\cdot \omega_{z}\cdot \Delta \omega_{y}\right)\frac{t^{4}}{24}$
$\Delta z_{3}$	$\Delta z_{3}=\left(f_{x}\cdot \omega_{z}\cdot \Delta \omega_{x}+f_{y}\cdot \omega_{z}\cdot \Delta \omega_{y}\right)\frac{t^{4}}{24}$
$\Delta x_{4}$	$\Delta x_{4}=VRW_{x}\cdot \frac{t^{\frac{3}{2}}}{\sqrt{3}}$
$\Delta y_{4}$	$\Delta y_{4}=VRW_{y}\cdot \frac{t^{\frac{3}{2}}}{\sqrt{3}}$
$\Delta z_{4}$	$\Delta z_{4}=VRW_{z}\cdot \frac{t^{\frac{3}{2}}}{\sqrt{3}}$
$\Delta x_{5}$	$\Delta x_{5}=\left(f_{z}\cdot ARW_{y}-f_{y}\cdot ARW_{z}\right)\frac{t^{\frac{5}{2}}}{2\sqrt{5}}$
$\Delta y_{5}$	$\Delta y_{5}=\left(f_{x}\cdot ARW_{z}-f_{z}\cdot ARW_{x}\right)\frac{t^{\frac{5}{2}}}{2\sqrt{5}}$
$\Delta z_{5}$	$\Delta z_{5}=\left(f_{y}\cdot ARW_{x}-f_{x}\cdot ARW_{y}\right)\frac{t^{\frac{5}{2}}}{2\sqrt{5}}$
$\Delta x_{6}$	$\Delta x_{6}=\left(f_{y}\cdot \omega_{x}\cdot ARW_{y}-f_{z}\cdot \omega_{z}\cdot ARW_{x}\right)\frac{t^{\frac{7}{2}}}{6\sqrt{7}}$
$\Delta y_{6}$	$\Delta y_{6}=-\left(f_{x}\cdot \omega_{x}\cdot ARW_{y}+f_{z}\cdot \omega_{z}\cdot ARW_{y}\right)\frac{t^{\frac{7}{2}}}{6\sqrt{7}}$
$\Delta z_{6}$	$\Delta z_{6}=\left(f_{x}\cdot \omega_{z}\cdot ARW_{x}+f_{y}\cdot \omega_{z}\cdot ARW_{y}\right)\frac{t^{\frac{7}{2}}}{6\sqrt{7}}$
$\Delta xv_{1}$	$\Delta xv_{1}=-\sin\psi \cdot \Delta v\cdot t$
$\Delta yv_{1}$	$\Delta yv_{1}=\cos\psi \cdot \Delta v\cdot t$
$\Delta zv_{1}$	$\Delta zv_{1}=\Delta V_{\zeta }\cdot t$
$\Delta xv_{2}$	$\Delta xv_{2}=-v_{N}\cdot \Delta \omega_{z}\frac{t^{2}}{2}$
$\Delta yv_{2}$	$\Delta yv_{2}=v_{E}\cdot \Delta \omega_{z}\frac{t^{2}}{2}$
$\Delta zv_{2}$	$\Delta zv_{2}=PRW_{z}\cdot \sqrt{t}$
$\Delta xv_{3}$	$\Delta xv_{3}=v_{N}\cdot \omega_{x}\cdot \Delta \omega_{y}\frac{t^{3}}{6}$
$\Delta yv_{3}$	$\Delta yv_{3}=-v_{E}\cdot \omega_{x}\cdot \Delta \omega_{y}\frac{t^{3}}{6}$
$\Delta xv_{4}$	$\Delta xv_{4}=-\sin\psi \cdot PRW\cdot \sqrt{t}$
$\Delta yv_{3}$	$\Delta yv_{3}=\cos\psi \cdot PRW\cdot \sqrt{t}$
$\Delta xv_{5}$	$\Delta xv_{5}=-v_{N}\cdot ARW_{z}\frac{t^{\frac{3}{2}}}{\sqrt{3}}$
$\Delta yv_{5}$	$\Delta yv_{5}=v_{E}\cdot ARW_{z}\frac{t^{\frac{3}{2}}}{\sqrt{3}}$
$\Delta xv_{6}$	$\Delta xv_{6}=v_{N}\cdot \omega_{x}\cdot ARW_{y}\frac{t^{\frac{5}{2}}}{2\sqrt{5}}$
$\Delta yv_{6}$	$\Delta yv_{6}=-v_{E}\cdot \omega_{x}\cdot ARW_{y}\frac{t^{\frac{5}{2}}}{2\sqrt{5}}$

## Appendix B

From the basic equations of inertial navigation by Taylor series expansion, it is possible to obtain systematic errors for SINS; for this we first calculate the derivatives of expressions (1):

(A1) $$\begin{array}{l} \ddot{\varphi }=\frac{{\dot{v}}_{N}}{R+h};\\ \ddot{\lambda }=\frac{{\dot{v}}_{E}}{\left(R+h\right)\cos\varphi };\\ \ddot{h}={\dot{v}}_{\varsigma }, \end{array}$$

Derivatives ${\dot{v}}_{N};{\dot{v}}_{E};{\dot{v}}_{U}$ are found from expressions (2) and (3) and are substituted into (A1)

(A2) $$\begin{array}{l} \ddot{\lambda }\left(R+h\right)\cos\varphi =f_{E}-\left(\frac{v_{E}v_{U}}{R}-\frac{v_{N}v_{U}}{R}tg\varphi +2\left(v_{\varsigma }\Omega \cos\varphi -v_{N}\Omega \sin\varphi \right)\right);\\ \ddot{\varphi }\left(R+h\right)=f_{N}-\left(\frac{v_{N}v_{U}}{R}+\frac{v_{E}^{2}}{R}tg\varphi +2v_{E}\Omega \sin\varphi -R\Omega^{2}\sin\varphi \cos\varphi \right);\\ \ddot{h}=f_{U}-\left(\frac{v_{N}^{2}}{R}-\frac{v_{E}^{2}}{R}-2v_{E}\Omega \cos\varphi -R\Omega^{2}\cos^{2}\varphi +g\right). \end{array}$$

From the matrix Equation (5) we obtain the desired projections $f_{E},f_{N},f_{U}$:

(A3) $$\begin{array}{l} f_{E}=c_{11}f_{x}+c_{12}f_{y}+c_{13}f_{z},\\ f_{N}=c_{21}f_{x}+c_{22}f_{y}+c_{23}f_{z},\\ f_{U}=c_{31}f_{x}+c_{32}f_{y}+c_{33}f_{z}. \end{array}$$

Projections $f_{E},f_{N},f_{U}$ are substituted into Equation (A2), which are then presented as follows:

(A4) $$\begin{array}{l} \ddot{\lambda }\left(R+h\right)\cos\varphi =F_{\lambda }\left(c_{11},c_{12},c_{13},f_{x},f_{y},f_{z}\right);\\ \ddot{\varphi }\left(R+h\right)=F_{\varphi }\left(c_{21},c_{22},c_{23},f_{x},f_{y},f_{z}\right);\\ \ddot{h}=F_{h}\left(c_{31},c_{32},c_{33},f_{x},f_{y},f_{z}\right), \end{array}$$

where the functions *F_φ_*, *F_λ_*, and *F_h_* are right-hand sides of Equation (A2).

We will look for systematic errors of SINSs, which depend on systematic errors of accelerometers and gyroscopes, by decomposition of Taylor ratios into a series (A4):

(A5) $$\begin{array}{l} \Delta \ddot{\lambda }\left(R+h\right)\cos\varphi =\frac{\partial F_{\lambda }}{\partial c_{11}}\Delta c_{11}+\frac{\partial F_{\lambda }}{\partial c_{12}}\Delta c_{12}+\frac{\partial F_{\lambda }}{\partial c_{13}}\Delta c_{13}+\frac{\partial F_{\lambda }}{\partial f_{x}}\Delta f_{x}+\frac{\partial F_{\lambda }}{\partial f_{y}}\Delta f_{y}+\frac{\partial F_{\lambda }}{\partial f_{z}}\Delta f_{z};\\ \Delta \ddot{\varphi }\left(R+h\right)=\frac{\partial F_{\varphi }}{\partial c_{21}}\Delta c_{21}+\frac{\partial F_{\varphi }}{\partial c_{22}}\Delta c_{22}+\frac{\partial F_{\varphi }}{\partial c_{23}}\Delta c_{23}+\frac{\partial F_{\varphi }}{\partial f_{x}}\Delta f_{x}+\frac{\partial F_{\varphi }}{\partial f_{y}}\Delta f_{y}+\frac{\partial F_{\varphi }}{\partial f_{z}}\Delta f_{z};\\ \Delta \ddot{h}=\frac{\partial F_{h}}{\partial c_{31}}\Delta c_{31}+\frac{\partial F_{h}}{\partial c_{32}}\Delta c_{32}+\frac{\partial F_{h}}{\partial c_{33}}\Delta c_{33}+\frac{\partial F_{h}}{\partial f_{x}}\Delta f_{x}+\frac{\partial F_{h}}{\partial f_{y}}\Delta f_{y}+\frac{\partial F_{h}}{\partial f_{z}}\Delta f_{z}, \end{array}$$

where Δλ is a longitude determination error; Δφ is a latitude determination error; Δ*h* is an altitude determination error; $\Delta \omega_{x},\Delta \omega_{y},\Delta \omega_{z}$ are biases or drifts of gyroscopes; $\omega_{x},\omega_{y},\omega_{z}$ are the vehicle’s angular velocity projections onto the vehicle reference frame, measured by three gyroscopes; and *t* is a time. Here $\Delta f_{E},\Delta f_{N},\Delta f_{U}$ are the biases of accelerometer projections onto the navigation reference frame and $\Delta f_{x},\Delta f_{y},\Delta f_{z}$ are the biases of accelerometer projections onto the vehicle’s reference frame. Values $\Delta f_{E},\Delta f_{N},\Delta f_{U}$ and $\Delta f_{x},\Delta f_{y},\Delta f_{z}$ are related by relation (5); *t* denotes time.

After double integration, we obtain expressions for systematic errors of SINS:

(A6) $$\begin{array}{l} \Delta \lambda =\frac{1}{R\cos\varphi }\left[\Delta f_{E}\frac{t^{2}}{2}+\left(f_{z}\cdot \Delta \omega_{y}-f_{y}\cdot \Delta \omega_{z}\right)\frac{t^{3}}{6}+\left(f_{y}\cdot \omega_{x}\cdot \Delta \omega_{y}-f_{z}\cdot \omega_{z}\cdot \Delta \omega_{x}\right)\frac{t^{4}}{24}+\ldots \right];\\ \Delta \varphi =\frac{1}{R}\left[\Delta f_{N}\frac{t^{2}}{2}+\left(f_{x}\cdot \Delta \omega_{z}-f_{z}\cdot \Delta \omega_{x}\right)\frac{t^{3}}{6}-\left(f_{x}\cdot \omega_{x}\cdot \Delta \omega_{y}+f_{z}\cdot \omega_{z}\cdot \Delta \omega_{y}\right)\frac{t^{4}}{24}+\ldots \right];\\ \Delta h=\Delta f_{U}\frac{t^{2}}{2}+\left(f_{y}\cdot \Delta \omega_{x}-f_{x}\cdot \Delta \omega_{y}\right)\frac{t^{3}}{6}+\left(f_{x}\cdot \omega_{z}\cdot \Delta \omega_{x}+f_{y}\cdot \omega_{z}\cdot \Delta \omega_{y}\right)\frac{t^{4}}{24}+\ldots , \end{array}$$

**Random errors of SINS**

The gyroscopes and accelerometers are characterized by random errors of the Angular Random Walk (ARW) [deg/$\sqrt{h}$] and Velocity Random Walk (VRW) [m/s/$\sqrt{h}$] types, respectively. Unlike the work in Ref. [23], where the errors of the SINS were represented by transfer functions, we will conduct research in the time domain.

If the input of the system is exposed to stationary white noise with spectral density *S*_0_ = *const*, then the variance or square of the standard deviation at the output of the system is determined by the dependence of the [23]

(A7) $$\sigma^{2}=S_{0}\int_{0}^{t}k^{2}(\tau )d\tau ,$$

where *k(τ)* is weighing function or pulse transient function of the system.

The relationship between the weight function of the system *k(t)* and its transient response *h(t)* is known [24]:

(A8) $$k(t)=\frac{dh(t)}{dt}.$$

Let us evaluate the effect of white noise of accelerometers with the VRM^2^ spectral function on the standard deviation of SINS error. According to expression (7) or (A6), the transient response of accelerometers is

(A9) $$h_{a}(t)=\frac{t^{2}}{2}.$$

Differentiating the right side *h_a_(t)* according to the expression (A8), the weight function of the accelerometers are determined:

(A10) $$k_{a}(t)=t.$$

In accordance with (A7), we determine the variance of the error due to the noise of the accelerometers:

(A11) $$\sigma_{a}^{2}=VRW^{2}\int_{0}^{t}t^{2}dt.$$

By calculating the integral and finding the square root, we get the standard deviation of the SINS error due to the noise of the accelerometers:

(A12) $$\sigma_{a}=VRW\frac{t^{\frac{3}{2}}}{\sqrt{3}}.$$

Similarly, let us find the standard deviations of the SINS error due to the noise of the gyroscopes:

(A13) $$\sigma_{g1}=ARW\frac{t^{\frac{5}{2}}}{2\sqrt{5}};\;\sigma_{g2}=ARW\frac{t^{\frac{7}{2}}}{6\sqrt{7}}.$$

Using this method, based on expression (9), we obtain the standard deviations of the errors of the VAN method due to the noise of the velocity meter PRW and the noise of the gyroscopes ARW:

(A14) $$\sigma_{v}=PRW\sqrt{t};\;\sigma_{vg1}=ARW\frac{t^{\frac{3}{2}}}{\sqrt{3}};\;\sigma_{vg2}=ARW\frac{t^{\frac{5}{2}}}{2\sqrt{5}}.$$
